# Ecological and Social Dimensions of Human–Bear Coexistence in Nepal's Gaurishankar Conservation Area

**DOI:** 10.1002/ece3.73776

**Published:** 2026-05-31

**Authors:** Shreyashi Bista, Nani Raut, Suman Shree Neupane, Morten Odden, Madhu Chetri

**Affiliations:** ^1^ Department of Environmental Science and Engineering, School of Science Kathmandu University Kathmandu Nepal; ^2^ University of South Florida Tampa Florida USA; ^3^ Faculty of Applied Ecology, Agricultural Sciences and Biotechnology University of Inland Norway Elverum Norway; ^4^ National Trust for Nature Conservation (NTNC) Khumaltar Nepal

**Keywords:** Asiatic black bear, conservation, habitat suitability, local perception, MaxEnt, ordered logistic regression

## Abstract

The increasing number of human‐black bear interactions in Nepal's Himalayas threatens both livelihoods and bear safety. Understanding human‐black bear coexistence has become increasingly important due to rising conflicts driven by habitat overlap, negative perceptions, and habitat degradation. Given the limited studies on the Asiatic Black bear in Nepal, particularly in the Gaurishankar Conservation Area, our research provides essential insights into its distribution, conflict patterns, and community perceptions. This study assessed the habitat suitability of Asiatic black bears and human‐black bear coexistence in the Gaurishankar Conservation Area, Nepal, using integrated ecological modelling and social analysis. We collected species presence data (*n* = 173) between September and December 2023 and analysed them using the MaxEnt algorithm. Additionally, we surveyed 188 households to assess the nature of conflicts and local perceptions toward black bears. The MaxEnt model performed well (AUC = 0.83 training; 0.73 validation), identifying 805.9 km^2^ (35.4%) as suitable habitat, primarily at elevations between 1000 and 3000 m. Habitat suitability was positively associated with forest diversity, precipitation during the driest quarter, and moderate human presence but declined sharply with increasing elevation and proximity to roads. Among the survey respondents (*n* = 188), 86% indicated that human‐bear conflict was increasing. Ordered logistic regression of perception data revealed that gender, ethnicity, elevation, and perceptions of ecological value and threats significantly influenced attitudes toward black bears (Pseudo *R* ^2^ = 0.57). Indigenous and female respondents reported higher perceived conflict, likely reflecting greater exposure to forest‐edge livelihood activities such as fuelwood collection, fodder gathering, and livestock care, whereas respondents recognising the ecological importance of black bears expressed greater tolerance. The study underscores that black bear conservation in Nepal requires both ecological and social interventions. Habitat conservation, coupled with community‐based awareness, livelihood support, and conflict mitigation programmes, is essential to foster long‐term coexistence.

## Graphical abstract

We modelled habitat suitability for Asiatic black bears in Nepal's Gaurishankar Conservation Area and combined it with community surveys to understand human–bear coexistence. Our results show that bear habitat is concentrated between 1000 and 3000 m and that conflict is rising in forest‐edge communities. The study highlights the need for habitat protection and community‐focused mitigation strategies.

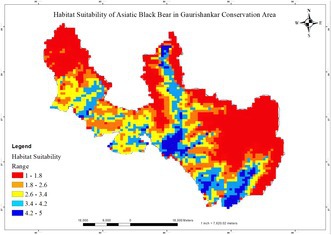

## 1 Introduction

The relationship between humans and wildlife is becoming increasingly strained as expanding human activities continue to encroach upon natural habitats (Narayan and Rana 2023). In Nepal's mountainous regions, which are rich in biodiversity and ecological significance, local communities are particularly vulnerable due to their heavy dependence on natural resources (Bista and Aryal 2013; Adhikari et al. 2018; Baral et al. 2021). This dependence has led to an increasing spatial overlap between humans and wildlife (Narayan and Rana 2023). Coexistence between humans and wildlife represents a critical conservation challenge, particularly when involving large carnivores, where interactions are complex for both humans and the species involved (Henle et al. 2008; Redpath et al. 2013; Van Eeden et al. 2018). Among the many species involved in human‐wildlife conflict, the Asiatic black bear ( *Ursus thibetanus* ) has emerged as one of the most conflict‐prone in Nepal's mountain regions due to its frequent involvement in crop raiding, livestock depredation, and attacks on humans (Chetri 2013; Awasthi and Singh 2015; Baral et al. 2021; Rawal et al. 2024). Rapid population growth, expanding infrastructure, and forestland urbanisation have resulted in habitat shrinkage and fragmentation, further escalating human‐black bear conflicts (Don Carlos et al. 2009; Escobar et al. 2015). Understanding how communities perceive and interact with Asiatic black bears is therefore essential for promoting coexistence, conserving biodiversity, and maintaining ecological balance (Hwang and Garshelis 2007). The rise in conflicts with humans is largely linked to habitat loss and degradation (Huygens et al. 2003; Charoo et al. 2011), which have contributed to population declines. Research on these issues tends to be less comprehensive in developing nations such as India and Nepal, where fatal encounters with bears occur more frequently (Chauhan 2003).

The Asiatic black bear (hereafter referred to as the black bear) is a species of the order Carnivora and family Ursidae, and one of the eight bear species found worldwide. It was categorised as Vulnerable under A2cd criteria worldwide on the International Union for Conservation of Nature (IUCN) Red List of Threatened Species (Garshelis and Steinmetz 2020). It is an omnivore whose distribution largely depends upon food accessibility, sufficient habitat resources, and levels of human pressure (Aryal 2011; Aryal et al. 2012). Although there are no assessments of the black bear population, it is estimated that fewer than 50,000 individuals remain in the wild (Garshelis and Steinmetz 2020). In Nepal, the species is listed as endangered on the National Red List of Mammals, with an estimated 500 individuals (Jnawali et al. 2011; Garshelis and Steinmetz 2020). The species typically inhabits mixed temperate oak forests, particularly those dominated by *Quercus semecarpifolia* (Chetri 2013). In central Nepal, black bears are found between elevations of 1600 and 3200 m (Bista and Aryal 2013), though in some regions they prefer 2500–3000 m, with an upper limit extending to 4300 m (Garshelis et al. 2016; Ali et al. 2017). Encounters resulting in human injuries caused by black bears are the most common type of human‐bear conflict in Nepal's mid‐hill regions (Bista and Aryal 2013; Baral et al. 2021). Between 2010 and 2014, black bears accounted for 12% of all wildlife encounters resulting in human death or injury (Bista et al. 2018). Moreover, in the Gaurishankar Conservation Area (GCA), it was responsible for 59 human‐wildlife conflict (HWC) incidents, resulting in the deaths of 87 livestock in 2021 (NTNC 2021). Additionally, four people (three males and one female) sustained serious injuries caused by a black bear (NTNC 2021). Respondents' attitudes toward the species were largely negative, with 80% expressing unfavourable views (Awasthi and Singh 2015). Given the increasing conflict and the continued loss of habitat, this study seeks to provide critical insights to support adaptive management strategies that balance ecological conservation with the needs of local communities. Understanding the distribution of large mammals is fundamental for effective management, as it helps conservationists and policymakers identify and prioritise areas for targeted conservation actions (Lu et al. 2012; Kichloo and Sharma 2021). Ecological niche modelling serves as a reliable and practical tool for mapping potential species ranges, guiding field research, and defining priority zones for conservation planning (Anderson and Martínez‐Meyer 2004; Bhattacharya et al. 2025).

Across the Himalayan and adjoining mountain regions, studies on black bears have documented strong associations between bear occurrence and forested mid‐elevation habitats, food availability, climate, terrain, and human disturbance, while also highlighting frequent conflict linked to crop raiding, livestock depredation, and encounters near settlements (Sathyakumar and Choudhury 2007; Charoo et al. 2011; Bista and Aryal 2013; Zahoor et al. 2021; Cheng et al. 2025). In Nepal, existing research has mainly focused on conflict incidence, local attitudes, elevational distribution, and habitat use in selected protected areas and mid‐hill landscapes (Chetri 2013; Bista and Aryal 2013; Bista et al. 2018; Rawal et al. 2024), leaving some geographic gaps in understanding black bear distribution and coexistence dynamics. Studies from India and China, together with broader Hindu Kush Himalayan analyses encompassing Bhutan, similarly show that black bear occurrence is shaped by interacting ecological and anthropogenic factors, but that these relationships remain highly context‐specific across landscapes (Sathyakumar and Choudhury 2007; Charoo et al. 2011; Zahoor et al. 2021; Cheng et al. 2025). This makes site‐level assessments essential, particularly in understudied conservation areas such as Gaurishankar, where habitat suitability, conflict patterns, and community perceptions have not previously been examined in an integrated framework.

Species distribution models (SDMs) are widely used to estimate potential species distributions by relating occurrence records to environmental and anthropogenic predictors (Guisan and Thuiller 2005). In conservation research, SDMs are particularly valuable for identifying suitable habitat, forecasting areas of human‐wildlife overlap, guiding field surveys, and prioritising management interventions (Guisan and Zimmermann 2000; Guisan and Thuiller 2005; Merow et al. 2014). Species distribution modelling offers a means to estimate the potential occurrence of species in areas lacking biological survey data and has therefore become an essential tool in conservation planning (Guisan and Zimmermann 2000; Loiselle et al. 2003). Among SDM approaches, MaxEnt has been widely applied because it performs well with presence‐only data and remains robust when absence data are unavailable, as is often the case for elusive mammals in mountain landscapes (Phillips et al. 2006). In this study, we used the Wallace EcoMod platform (Kass et al. 2018), which provides a transparent and reproducible workflow for ecological niche modelling. Such approaches are especially relevant in conflict‐prone landscapes because they help identify habitat patches and overlap zones where conservation action and conflict‐mitigation measures may need to be prioritised.

This study aims to integrate ecological niche modelling and socio‐economic analysis to better understand habitat suitability, human‐black bear conflict patterns, and local perceptions in the GCA, Nepal. Specifically, we aim to (i) model the spatial distribution and habitat suitability of the black bear using environmental and anthropogenic predictors; (ii) quantify the nature and drivers of human‐black bear conflicts; and (iii) examine how socio‐demographic factors influence community perceptions toward the species.

## 2 Materials and Methods

### 2.1 Study Area

**FIGURE 1:**
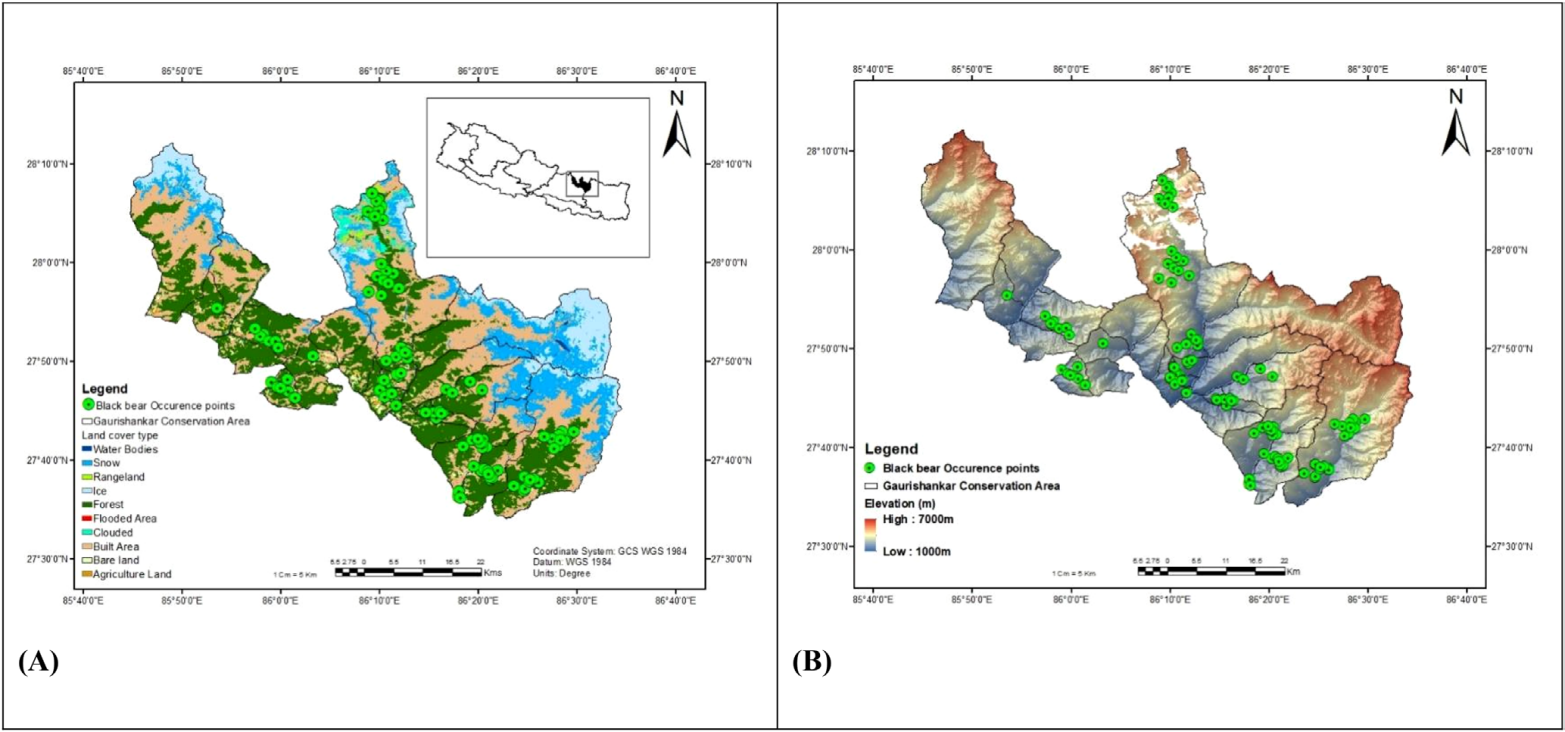
Study area maps of Gaurishankar Conservation Area (GCA), Nepal. (A) Land cover and black bear occurrence points in the study area. (B) Digital elevation model (DEM) and black bear occurrence points in GCA.

The GCA was established as a Conservation Area by the Nepalese government in January 2010. It covers an area of 2179 km^2^ encompassing 8 rural municipalities and 2 municipalities, and spreads over three districts: Dolakha, Ramechhap and Sindhupalchok (Figure 1). It is positioned between 85°46.8′ and 86°34.8′ east longitude and 27°34.2′ to 28°10′ north latitude (NTNC 2009) and lies between two important mountain national parks: Langtang National Park (LNP) in the west and Sagarmatha National Park (SNP) in the east. Around 67,000 people of diverse ethnic groups are living within the GCA, comprising 50.4% males and 49.5% females, with the largest group being Janajatis (Indigenous), who make up 72.59% of the population, followed by Chhetri at 15.71% and Dalits at 8.01% (Sharma et al. 2018). The remaining population includes Brahmins, Thakuris, and Sanyasis. At higher elevations, ethnic groups of Tibetan origin, such as Sherpas and Yolmopa, are predominant, while the lower hilly areas are mostly inhabited by Tamang, Thami, Sunuwar, and some other unique ethnic groups like Surel and Jirel. Buddhism and Hinduism are the primary religions practised by the local population (Sharma et al. 2018).

The vegetation cover varies significantly, with forests and shrubs dominating 44.5% of the area, followed by cultivated land at 8.8%, grasslands at 8.6%, glaciers at 2.8%, and barren ground and other terrain comprising the remaining 35.3% (NTNC 2023). The GCA has a diverse range of 16 major vegetation types, including the *Pinus roxburghii* forest, *Schima‐Castanopsis* Forest, Alnus forest, *Pinus wallichiana* forest, *Pinus patula* forest, Rhododendron Forest, *Quercus lanata* forest, Lower Temperate Oak Forest (*Quercus Semicarpifolia* Forest), Lower Temperate Mixed Broad‐Leaved Forest, Abies forest, and Upper Temperate Mixed Forest (Birch Forest). The area is rich in biodiversity, harbouring 722 wild plant species, 77 mammal species, 27 fish species, 12 amphibian species, 29 reptile species, and 271 bird species (Chetri et al. 2022; NTNC 2023). It also provides habitats for endangered and threatened species such as the Red Panda ( *Ailurus fulgens* ), Musk Deer ( *Moschus chrysogaster* ), Snow Leopard ( *Panthera uncia* ), black bear, and several other important smaller mammalian taxa (Shrestha and Meng 2014). The area serves as a crucial catchment for several major rivers, including the Khimti, Bhotekoshi, Sunkoshi, and Tamakoshi, which supply water to some of the country's largest hydropower projects. Notably, the region is also known for its glacial lakes, with Tsho Rolpa gaining particular attention due to concerns about its potential for glacial lake outburst floods (GLOFs) (NTNC 2021).

### 2.2 Data Collection

#### 2.2.1 Species Presence Data and Habitat Mapping

We collected black bear presence coordinates in the GCA from September to December 2023. Surveys were conducted opportunistically along existing forest roads, livestock herding routes, and accessible forest areas identified through participatory resource mapping with residents. Although fixed transects were not established, efforts were made to cover a range of habitat types and elevational zones within the study area. We recorded both direct evidence (direct sightings) and indirect evidence (faeces, tree bedding, claw marks, footprints, hair, and feeding signs) of black bears, together with associated covariates such as habitat type, food resources, and topographic features, using a GPS. In total, 173 presence locations were obtained during the survey. Potential habitat areas were also identified through informal interviews with residents and conservation area staff.

#### 2.2.2 Human‐Black Bear Conflict and Perception

An interview‐based questionnaire survey was conducted from January 2023 to November 2023 to document human‐black bear conflict experiences and local perceptions in the GCA. This approach has been widely used in comparable human‐wildlife conflict studies and has been shown to provide valuable insights (e.g., Goursi et al. 2021; Ahmad et al. 2022). After consultation with authorities (NTNC officials and GCA office staff), Bigu Rural Municipality was selected because it was identified as an area with high reporting of human‐black bear interactions. The municipality comprises nine wards, and all wards were included in the survey to ensure spatial coverage. Bigu Rural Municipality was also considered appropriate for the social survey because it lies near black bear occurrence areas, and a suitable habitat was identified within the GCA.

An official household list was obtained, and a target of approximately 20 respondents per ward was planned. Within each ward, field visits were conducted in consultation with ward leaders, tourist guides, and local conservationists to identify areas with frequent human‐black bear interactions. From these identified localities, households were purposively selected from the household list, and respondents from each household were approached for interviews. When respondents were unavailable at home, interviews were conducted in agricultural fields during cropping periods to ensure adequate coverage of actively farming households.

In addition to the ward‐level sampling, eight respondents from Orang (*n* = 3), Bulung (*n* = 2), and Lamabagar (*n* = 3) were purposively included because they had experienced recent human injury incidents or livestock depredation, as reported by ward leaders. The survey was therefore purposive and household‐based rather than proportionately stratified by demographic categories such as gender, age, education, or occupation. However, inclusion of all wards and multiple conflict‐prone localities allowed participation from diverse socio‐economic groups within the municipality (see Table 4).

In total, 188 respondents were interviewed using semi‐structured questionnaires. The questionnaire comprised 42 questions covering demographic information; agricultural and livestock data, including crop losses and depredation; information on black bears and their locations; records of human injuries; compensation information; and local perceptions of black bears and conservation. For understanding local perceptions, we focused on the independent variables gender, age, education level, livestock units, landholdings, whether respondents had seen a black bear, the number of black bears seen, time since the last black bear sighting, and distance to forest areas (Table 1). Before each interview, the purpose of the study was explained to participants and informed verbal consent was obtained. Consent procedures were facilitated with the support of NTNC colleagues and ward officers in each ward. Participation was voluntary, and respondents were informed that they could decline to answer questions or withdraw from the interview at any time. The composition of the final sample across key socio‐economic categories is presented in Table 4 to provide transparency regarding respondent characteristics.

**TABLE 1:** Description of the variables used in perception analysis of black bear in Gaurishankar Conservation Area, Nepal.

Socio‐economic indicators	Description
Conflict level	
Decreased	Respondents were asked about the level of conflict at the current time
Same	
Increased	
Elevation	Elevation of the households of the respondents
Sex	
Male	A binary variable that represents the gender of HH (female = 1, otherwise 0)
Female	
Ethnicity	
Indigenous	A categorical variable that represents the ethnicity of the respondent (Indigenous = 1, Brahmin/Chhetri/Thakuri = 2, Dalit = 3)
Bramhin/Chhetri/Thakuri	
Dalit	
Education	
Uneducated	An Ordinal Scale that represents the education of the respondents: 1 = uneducated, 2 = primary, 3 = secondary, 4 = higher secondary education, and 5 = university
Primary (up to 5 years)	
Secondary	
Higher secondary	
University	
Age	The age of the respondent is calculated from their date of birth in years
Human_injury_death	A binary variable that represents death or injury caused by a black bear (yes = 1, otherwise = 0)
Landholdings	Total land owned by the HH (in hectares)
Livestock unit (LSU)	Cumulative livestock unit from the absolute number and type possessed by HH. The estimated livestock numbers were converted into livestock units (LSU) using standard conversion factors applied in Nepal, where one adult buffalo equals 1 LSU, cattle equal 0.7 LSU, and goats equal 0.2 LSU (Thapa and Paudel 2000; Agrawal and Gupta 2005)
Bear seen	
Yes	A binary variable that represents if a respondent has seen a bear in the past 5 years (seen = 1, otherwise 0)
No	
Number of bears seen	Total number of bears seen by the respondent
Time	Number of months passed since the respondent had seen the last bear
Distance	Nearest walking distance to the forest areas (in metres)
Ecological value of the bear	A binary variable that represents if respondents think black bear is ecologically valuable (yes = 1, otherwise = 0)
Bear threat to crop/livestock	A binary variable that represents if respondents think black bears are a threat to their crops and livestock (yes = 1, otherwise = 0)

### 2.3 Data Analysis

#### 2.3.1 Habitat Suitability Mapping

For habitat suitability modelling, 13 environmental and 3 anthropogenic variables (Table 2) were selected for use with species occurrence points. These included 9 global bioclimatic variables sourced from the WorldClim historical database, which gathered data from 4000 stations at a 1 km spatial resolution. In species distribution modelling, removing highly correlated variables helps avoid redundancy and ensures more accurate, unbiased predictions (Merow et al. 2013). Multicollinearity among these variables was assessed using the Variance Inflation Factor (VIF) (Zuur et al. 2010) and the Pearson correlation coefficient (r), with a threshold of VIF < 5 and *r* < 0.7. Variables exhibiting high collinearity or redundancy were removed, and variables with low collinearity were retained (Table 3) to ensure statistical robustness and ecological clarity. We resampled the WorldClim bioclimatic rasters from approximately 1 km to 100 m in ArcGIS using the Resample tool with bilinear interpolation, which is recommended for continuous raster surfaces such as climatic variables. This was done to align the bioclimatic layers with the finer‐resolution topographic and anthropogenic predictors used in the model (Manzoor et al. 2018; Rather et al. 2020).

**TABLE 2:** Description of the environmental and anthropogenic variables used in habitat suitability of the black bear in Gaurishankar Conservation Area, Nepal.

Category	Variables	Unit	Source
Bio climatic	Annual mean temperature (bio1)	°C	WORLDCLIM
	Mean diurnal range (bio2)	°C	
	Isothermality (bio3)	°C	
	Temperature seasonality (bio4)	°C	
	Max temperature of warmest month (bio5)	°C	
	Annual precipitation (bio12)	mm	
	Precipitation of seasonality (bio15)	mm	
	Precipitation of driest quarter (bio17)	mm	
	Precipitation of coldest quarter (bio19)	mm	
Topographic and vegetation related	Distance to water	Metres	USGS
	Forest types	Categorical vegetation class	GEO FABRIK
	Land cover and land use	LULC	ICIMOD
	Elevation	Metres	GOOGLE EARTH
Anthropogenic	Distance to road	Degrees	Road distance was calculated using raster‐based Euclidean distance analysis and used as a continuous predictor in the MaxEnt model
	Distance to settlement	Degrees	Settlement distance was calculated using raster‐based Euclidean distance analysis and used as a continuous predictor in the MaxEnt model
	Livestock (cattle, sheep, goats)		Human and livestock variables were incorporated as raster‐based population layers representing the spatial distribution of human settlements and livestock abundance across the study area
	Human population		

**TABLE 3:** Final Variables retained in the model after removing collinear ones.

S.N.	Variable	VIF value
1	Bio4	2.31
2	Bio17	2.71
3	Foresttypes	1.39
4	Aspect	1.50
5	Cattle	2.95
6	Water	1.13
7	Road	1.74
8	Goat	1.34
9	Humanpopn	2.49
10	Settlement	2.75
11	Elevation	6.22

#### 2.3.2 SDM

We ran the MaxEnt model using the Wallace platform (Kass et al. 2018) in the R version 4.3.2 (R Core Team 2023) to predict the potential distribution of black bears in the GCA (Phillips et al. 2006). The Wallace EcoMod platform provides an interactive, reproducible environment for model calibration and evaluation. The MaxEnt algorithm is particularly effective when true absence data are unavailable (Su et al. 2018; Deb et al. 2019). We spatially thinned the occurrences at 1 km to reduce autocorrelation and sampling bias. Environmental and anthropogenic predictors (Table 3) were processed at a buffer resolution of 0.18 degrees, and 3000 background points were generated across the study area to characterise available environmental conditions in the GCA. This number was considered sufficient to capture environmental heterogeneity for model calibration while remaining computationally manageable. To minimise overfitting, spatial partitioning was applied using a checkerboard method (*k* = 2, aggregation factor = 2). Models were run with all feature classes (linear, quadratic, hinge, product: lqhp), a regularisation multiplier of 1, 2 and 3, and a multiplier step value of 1. Clamping was enabled to prevent extrapolation beyond training data ranges, and parallel processing was disabled to ensure reproducibility. Predictions were produced using the cloglog transformation of the 10th‐percentile training presence threshold to determine species presence (Escobar et al. 2015; Kabir et al. 2017). The final model was selected as the one with the lowest AICc while maintaining acceptable AUC and omission rate values. We used AUC as the primary evaluation metric because it is threshold‐independent and relatively less sensitive to prevalence, which makes it appropriate for a MaxEnt‐based presence‐background framework where true absence data were unavailable (Manel et al. 2001; McPherson et al. 2004; Franklin et al. 2009).

#### 2.3.3 Local Perceptions

We employed ordered logistic regression to explore how various factors influence local communities' perceptions of black bear habitat and population trends (Table 1). All quantitative analyses were carried out in R 4.1.1, principally by estimating an ordered logistic regression model with the *polr* function from the MASS package (Venables and Ripley 2002). The command name emphasises our model's proportional odds assumption by deriving it from logistic regression with proportional odds. *polr* specifies a regression model with response and predictors using *R*'s standard formula interface. We also set Hess = TRUE to make the model provide the observed information matrix from optimization, which is used to calculate standard errors.

## 3 Results

### 3.1 Habitat Suitability

#### 3.1.1 Variables Retained

A total of 10 predictor variables: Bio4, Bio17, forest types, aspect, cattle population, water sources, distance to roads, goat population, human population and settlement were retained for the final species distribution modelling of the black bear (Table 3). All selected variables exhibited VIF values below 3, indicating no significant multicollinearity and confirming their suitability for inclusion in the MaxEnt models.

Conversely, BIO1, BIO3, BIO5, BIO12, BIO15, BIO19, livestock (total), and sheep displayed high collinearity and were therefore excluded from the final model, with one exception. Elevation was deliberately retained, despite its statistical collinearity, because of its biological and ecological importance. Elevation directly influences climatic conditions (such as temperature and precipitation gradients), vegetation types, and habitat structure, which are critical for black bear distribution, so excluding it might result in the loss of an essential topographic gradient that defines black bear habitat suitability.

#### 3.1.2 Predicted Habitat Suitability of the Black Bear

**FIGURE 2:**
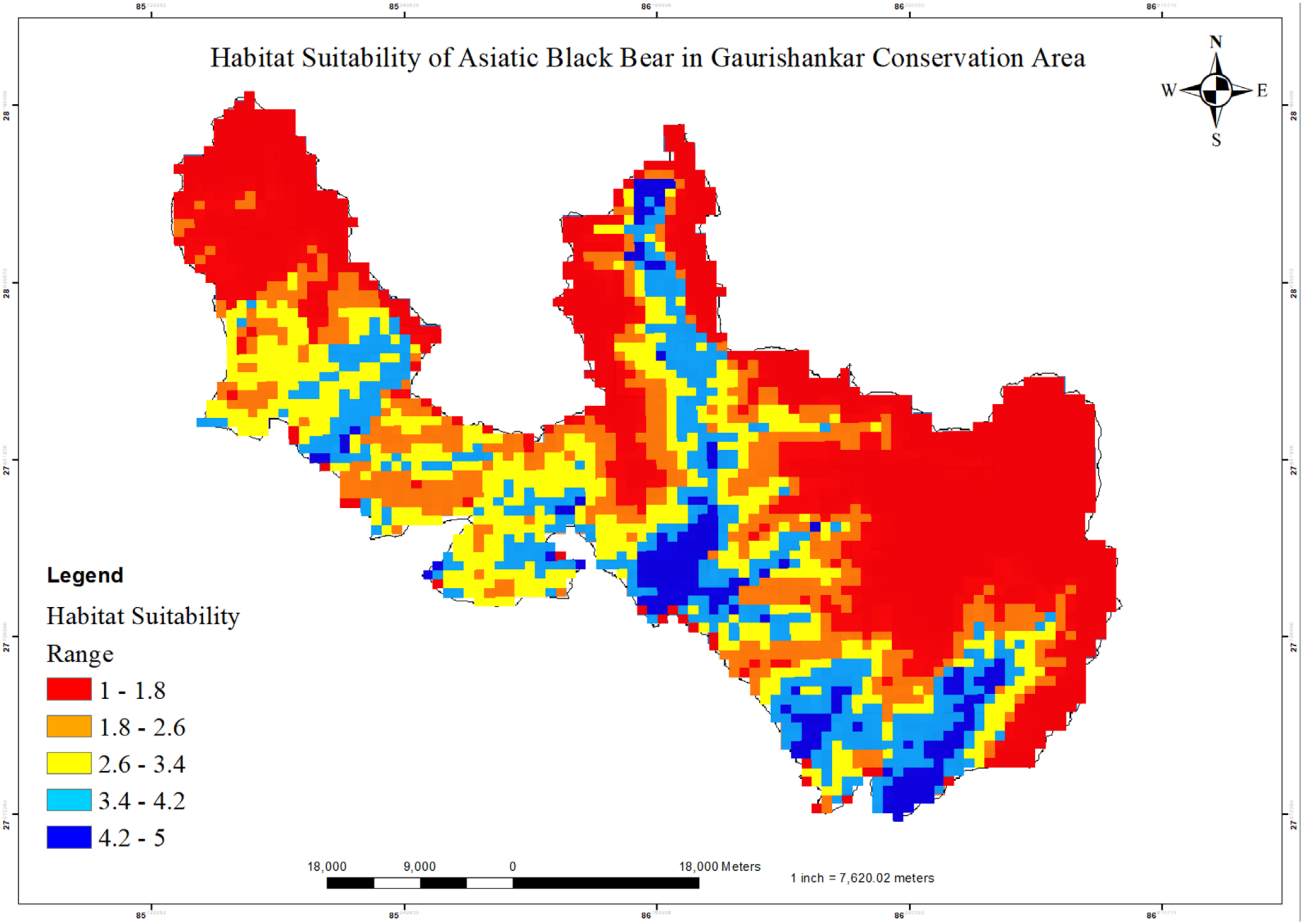
Predicted habitat suitability of the black bear in the Gaurishankar Conservation Area, Nepal, based on MaxEnt modelling.

Among all model configurations tested, the LQHP feature combination (Linear + Quadratic + Hinge + Product) with a regularisation multiplier (RM) = 3 was identified as the best‐performing model. This configuration achieved the lowest corrected Akaike information criterion (AICc = 1783.72; ΔAICc = 0), indicating the strongest statistical support among candidate models. The final model showed good predictive performance with minimal overfitting (average training AUC = 0.83; validation AUC = 0.73) (Figure 2).

**FIGURE 3:**
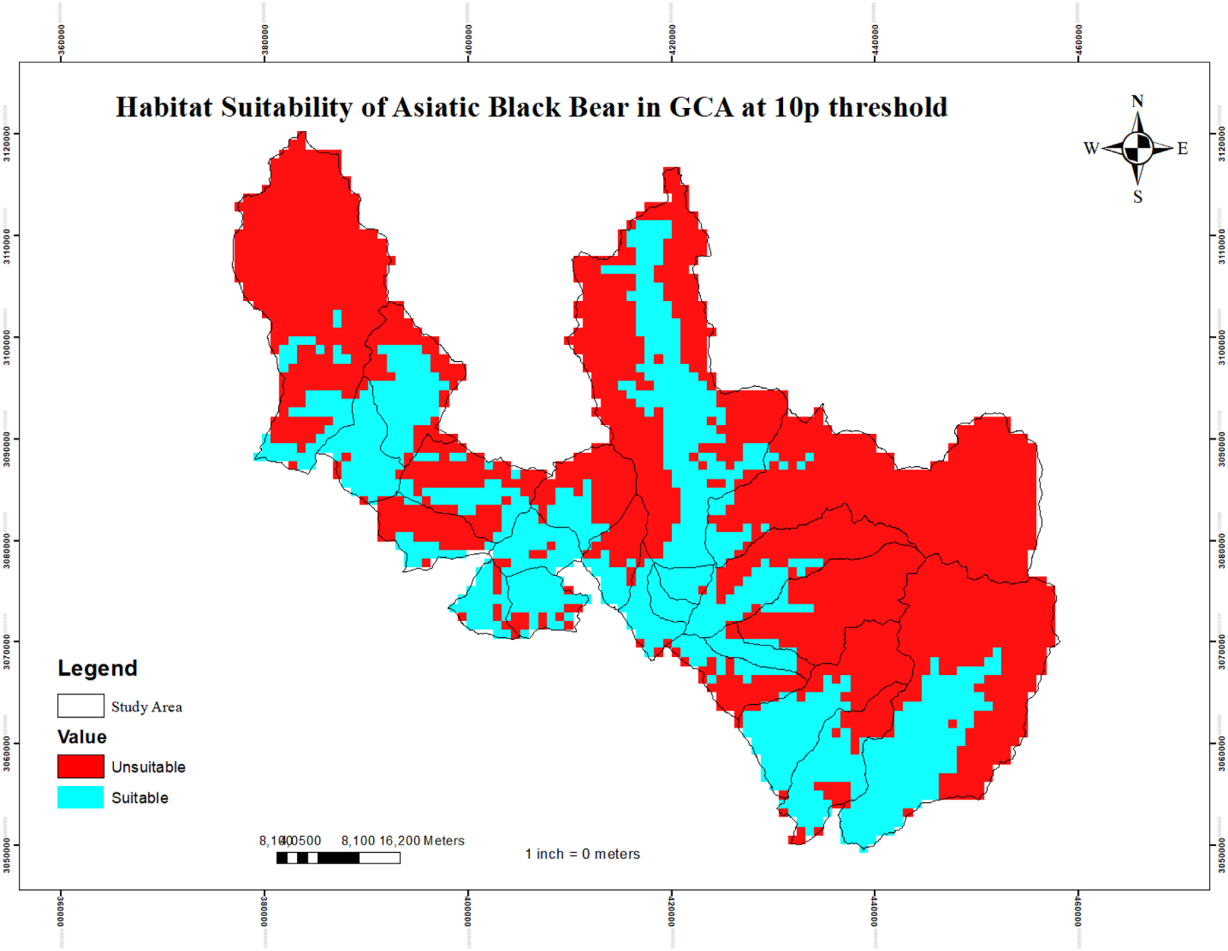
Binary habitat suitability map for the black bear in the Gaurishankar Conservation Area at the 10th percentile training presence (10p) threshold.

To convert the continuous MaxEnt output into a binary suitable/unsuitable map (Figure 3), we tested two commonly used thresholds: the minimum training presence (MTP = 0.03) and the 10th‐percentile training presence (10p = 0.17). The predicted suitable habitat thus obtained was 805.9 km^2^ (35.4% of the total area) (Figure 3). According to the results, Shyama, Chuchure, Gumdel, Lamabagar, Orang, and Bulung provide ideal habitats for black bears.

#### 3.1.3 Response to Environmental Variables

**FIGURE 4:**
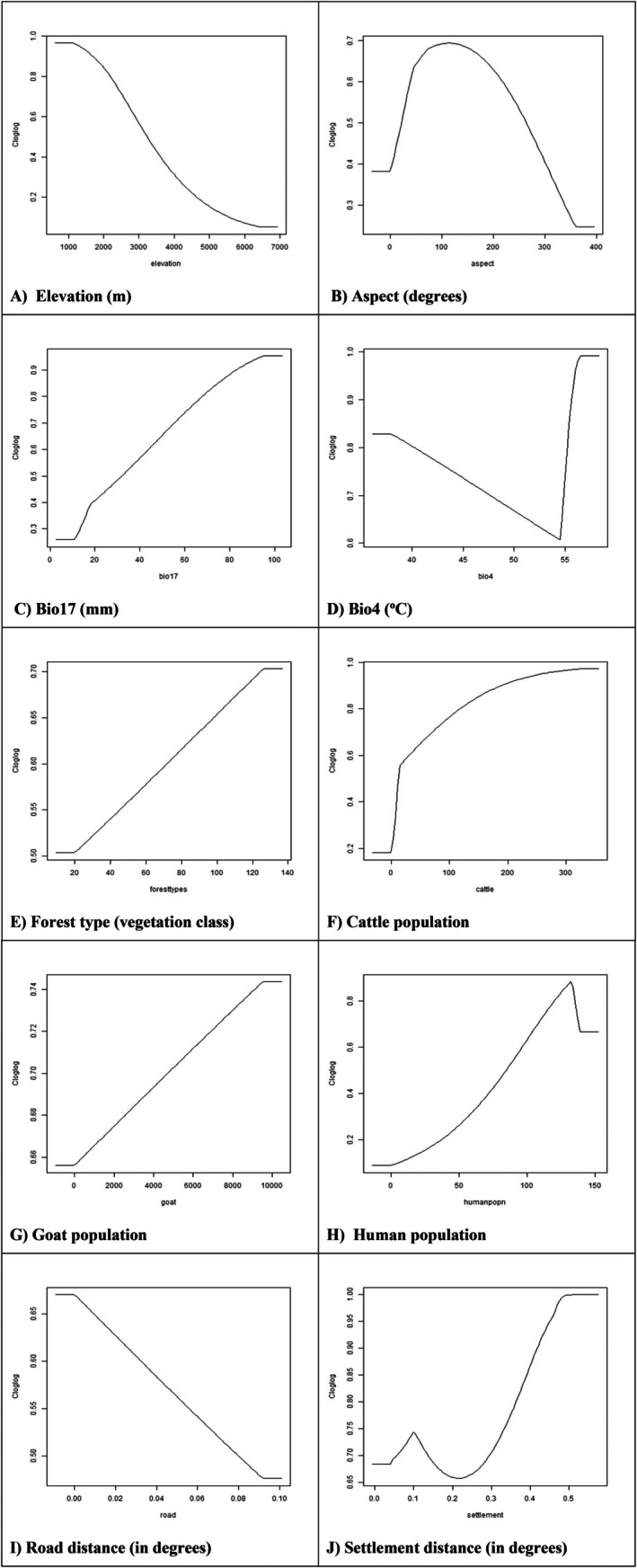
Response curves of environmental and anthropogenic variables influencing habitat suitability of Asiatic black bears in Gaurishankar Conservation Area, Nepal. (A) Elevation, (B) aspect, (C) precipitation of driest quarter (Bio17), (D) temperature seasonality (Bio4), (E) forest type, (F) cattle population, (G) goat population, (H) human population, (I) road distance, and (J) settlement distance.

The response curve for elevation shows that predicted habitat suitability (cloglog output) is highest at low elevations and decreases with increasing elevation. At 800–1500 m, the cloglog value is about 0.9–1.0, around 3000 m it is roughly 0.5–0.6, around 5000 m it is about 0.2, and above ~6500 m it is below 0.1 (Figure 4A). Habitat suitability varied with aspect, being highest on slopes with aspects of about 60°–160°, moderate near 0°–20°, and lowest on aspects above approximately 260°–360° (Figure 4B). Habitat suitability increased with dry‐season rainfall (Bio17), being lowest at values below about 10–15 mm and highest at values around 80–100 mm of precipitation in the driest quarter (Figure 4C). Habitat suitability was fairly high when temperature seasonality was low (around 37–40). It gradually becomes lower as seasonality increases to about 52–54, and then becomes very high again at the highest seasonality values (around 55–56) (Figure 4D). Habitat suitability differed among forest‐type categories (Figure 4E). In the study area, black bears were mainly associated with mid‐elevation temperate and lower temperate forest types, particularly *Schima‐Castanopsis*, *Quercus* spp. with *Arundinaria* spp., mixed broad‐leaved, and *Pinus wallichiana* forests. Among anthropogenic variables, habitat suitability increased with cattle numbers, rising at low cattle densities and then increasing more slowly and levelling off at higher densities (Figure 4F), and habitat suitability also increased with goat numbers, being lower at low numbers and higher at high numbers (Figure 4G). Habitat suitability increased with human population, being low at very low population values, highest at relatively high population values, and slightly lower again at the very highest values (Figure 4H). This pattern suggests that black bears may use forest–settlement or forest–agriculture interfaces, where habitat cover remains available, and supplementary food resources may occur. The decline at the highest population values likely reflects increasing disturbance and reduced habitat security in more densely populated areas. Habitat suitability was higher farther from roads and lower closer to roads, suggesting that black bears may avoid areas with greater road‐related disturbance (Figure 4I). Habitat suitability was moderate farther from settlements, declined at intermediate distances, and increased again near settlements, suggesting possible use of forest–settlement interface areas rather than direct preference for settlements (Figure 4J).

### 3.2 Human Black Bear Conflict and Perceptions

The demographic composition of the survey participants (*n* = 188) included 91 males (47%) and 97 females (53%). Among them, 43% identified as Indigenous (Tamang, Newar, Gurung, Sherpa, and Magar), 25% as Dalit, and 32% as Brahmin, Thakuri, or Chettri. Regarding education, 25% had primary education (up to grade 5), 20% had secondary education (grades 6–10), 5% higher secondary, 1% university‐level, and 49% had no formal education (Table 4). Although sampling was conducted at the household level rather than proportionately stratified by demographic categories, the resulting sample captured representation across gender, ethnicity, and education levels within the community.

**TABLE 4:** Socio‐economic characteristics of surveyed respondents (*n* = 188) in Bigu Rural Municipality, Gaurishankar Conservation Area, Nepal.

Socio‐economic indicators	Values
Conflict level	
Decreased	85.88%
Same	8.24%
Increased	5.88%
Gender	
Male	47%
Female	53%
Ethnicity	
Indigenous	43%
Bramhin/Chhetri/Thakuri	32%
Dalit	25%
Education	
Uneducated	49%
Primary education	26%
Secondary	20%
Higher secondary	4%
University	1%
Age	38.78 ± 10.58 (18–68)
Landholdings (in hectares)	0.41 ± 0.36 (0.05–3.1)
Livestock unit (LSU)	8.82 ± 7.65 (0–47)
Bear seen	
Yes	60%
No	40%
Number of bears seen	1.37 ± 0.69 (1–5)
Time	5.6 ± 4.69 (0–16)
Distance	200.1913

The average landholdings were 0.4 ha, and the calculated mean livestock unit was 8.82. About 60% of respondents reported having seen black bears, with an average of 1.37 black bears sighted. The mean time since the last black bear sighting was 5.6 months. Regarding conflict, 86% reported an increase, 8% a decrease, and 6% had no opinion, indicating that local people may be experiencing growing conflict in the study area (Table 4).

The ordered logistic regression results showed that male respondents were less likely to perceive an increase in conflict within GCA than female respondents (Table 5). The positive coefficient for elevation, with *p*‐value (6.04e^−08^), suggests that people at higher elevations were more likely to perceive increasing conflict. The positive coefficient for Indigenous status, with a significant *p*‐value (1.99e^−02^), indicates that Indigenous respondents were more likely than non‐Indigenous respondents to perceive increasing conflict. The ecological value of black bears had a strongly negative coefficient with a highly significant *p*‐value (6.99e^−13^), indicating that respondents who viewed black bears as ecologically important were less likely to perceive high conflict. Likewise, a positive coefficient for black bears as a threat to crops/livestock with *p*‐value (3.17e^−02^) indicates that respondents who perceived black bears as threats were more likely to perceive increasing conflict. The Pseudo *R* ^2^ value of 0.57 suggests that the model explains 57% of the variation in perceptions about black bear conflicts in GCA, indicating a good model fit (Table 5). The Pseudo *R* ^2^ value reflects the model's explanatory power, particularly useful in non‐linear models where conventional R‐squared measures are not applicable.

**TABLE 5:** Regression model for analysing people's perception.

Ordered logistic regression model of people's perception of the status of black bears in GCA				
Independent variables	Coefficient	SE	*t*‐value	*p*
Elevation	0.007	0.001	5.418	6.04e^−08^ ***
Ethnicity	2.225	0.956	2.328	1.99e^−02^ *
Education	0.092	0.484	0.189	8.49e^−01^
Sex (male)	−2.029	0.917	−2.213	2.69e^−02^ *
Age	−0.067	0.043	−1.561	1.19e^−01^
Human_injury_death	−1.121	1.034	−1.084	2.78e^−01^
Distance	0.003	0.006	0.542	5.87e^−01^
Livestock unit (LSU)*	0.003	0.057	0.053	9.58e^−01^
Landholdings (in hectares)	−0.062	0.045	−1.372	1.70e^−01^
Bear seen	−0.830	1.144	−0.726	4.68e^−01^
No. of black bear seen	−0.023	0.663	−0.034	9.72e^−01^
Time	−0.142	0.116	−1.22521	2.21e^−01^
Ecological value of bear	−4.138	0.576	−7.179	6.99e^−13^ ***
Bear threat to crop/livestock	3.304	1.538	2.148	3.17e^−02^ *

*Note:* Observations: 188. Log likelihood: −20.9327912715119. Pseudo *R* ^2^: 0.568687453115357. LR *χ* ^2^ (*p*): 55.1999511295243 (0.0000064).

* *p* < 0.1.

*** *p* < 0.01.

## 4 Discussion

The MaxEnt model performed well in predicting the habitat suitability of black bears, with a training AUC of 0.83 and a validation AUC of 0.73, indicating good model accuracy and predictive reliability. Similar AUC ranges have been reported by other ecological niche modelling studies (Kabir et al. 2017; Bai et al. 2018; Hameed et al. 2020; Hosni et al. 2020; Ahmadipari et al. 2021), and values above 0.7 are generally considered strong for MaxEnt models (Araújo and New 2007; Ancillotto et al. 2020). This consistency with earlier research confirms the robustness of the present model in capturing the ecological preferences of black bears (Warren and Seifert 2011). Using presence‐only data and selected environmental predictors, the model provided a reliable framework for mapping potential black bear habitats in the GCA. This is consistent with the broader effectiveness of MaxEnt in predicting species distributions across diverse taxa and regions, particularly when only limited sample data are available (Hernandez et al. 2006; Pearson et al. 2007; Baldwin 2009). Similar spatial modelling approaches have proven valuable for conservation planning and habitat management in other regions (Kabir et al. 2017; Farashi and Erfani 2018; Morovati et al. 2020; Ahmadipari et al. 2021; Su et al. 2021). Although predicted maps may contain some uncertainty, especially for generalist species like black bears (Jiménez‐Valverde et al. 2008), the high AUC and low omission rates in this study indicate strong model performance and reliability. Using the 10p threshold, the model identified 805.9 km^2^ as suitable habitat for the black bear. The MTP threshold represents the lowest predicted suitability value among training records and therefore minimises omission error but tends to produce overpredicted areas (Liu et al. 2005; Radosavljevic and Anderson 2014). The 10p threshold excludes the lowest 10% of presence records, reducing the influence of potential outliers or spatial uncertainty and yielding more ecologically meaningful predictions (Jiménez‐Valverde and Lobo 2007; Pearson et al. 2007). Because our study aims to inform conservation management, where minimising false positives is critical, we selected the 10p threshold for binary habitat classification.

The model highlighted that elevation, precipitation, temperature, and forest types are factors shaping habitat suitability. Black bears showed a clear preference for low‐ to mid‐elevation zones between 1000 and 3000 m, which are typically characterised by temperate climates and mixed forests. Suitability declined rapidly above 4500 m, suggesting that high alpine regions are less favourable. This pattern is consistent with studies from Nepal, where black bears have commonly been recorded between 1500 and 3500 m (Bista and Aryal 2013; Chetri 2013; Kadariya et al. 2018; Subedi et al. 2021), and from Pakistan, where they have likewise been reported mainly within 2000–3500 m (Ali et al. 2017; Waseem et al. 2020). Comparable evidence is also available from India, Bhutan, and China. In India, Sathyakumar (2001) and Sathyakumar and Choudhury (2007) noted that black bears are largely associated with temperate and subalpine forest zones and generally occur below about 4500 m. In Bhutan, Letro et al. (2020) recorded black bears between approximately 1218 and 4389 m in Jigme Singye Wangchuck National Park. In China, Cheng et al. (2025) reported habitat use beginning above 1000 m, indicating some regional variation in elevational distribution depending on local climatic and habitat conditions. Garshelis and Steinmetz (2020) and Bashir et al. (2018) further observed that, although the species occasionally enters alpine meadows, records above 4200 m remain uncommon. Together, these studies suggest that *Ursus thibetanus* is ecologically flexible across a broad elevational gradient, but is most strongly associated with low‐ to mid‐elevation temperate forests where food resources, cover, and denning opportunities are greatest (Escobar et al. 2015; Zahoor et al. 2021; Cheng et al. 2025). In the study area, black bears were mainly associated with mid‐elevation temperate and lower temperate forest types, particularly *Schima‐Castanopsis*, *Quercus* forests with *Arundinaria* understory, mixed broad‐leaved, and *Pinus wallichiana* forests. This pattern is broadly consistent with previous work from Nepal. In the southeastern Annapurna Conservation Area, most black bear signs were recorded between 1900 and 3100 m, and bears were mainly associated with *Schima wallichii*, *Quercus* forests with *Arundinaria* spp. (Bista and Aryal 2013). Similar patterns have been reported elsewhere, with black bears frequently associated with forested mid‐elevation habitats, especially temperate, mixed broad‐leaved, and other structurally complex mountain forest types (Sathyakumar 2001; Izumiyama and Shiraishi 2004; Bista and Aryal 2013; Ali et al. 2017).

The positive relationship between precipitation during the driest quarter (Bio17) and habitat suitability suggests that relatively moist conditions during the dry season may be important for black bear persistence. Even in otherwise seasonal mountain environments, dry‐season moisture likely helps maintain vegetation productivity, water availability, and food resources. Temperature seasonality (Bio4) was also an important predictor, indicating that black bear suitability is influenced by climatic variability across the year. In the present study, temperature‐ and precipitation‐related variables were among the most important predictors, suggesting that climate plays a key role in shaping black bear habitat suitability. These climatic variables are likely to influence black bears largely indirectly, through their effects on vegetation growth, forest structure, and the seasonal availability of food and cover. Water availability is fundamental to plant metabolic processes (Wang et al. 1998), and reduced precipitation can limit plant growth and productivity, particularly in moisture‐stressed environments (Khwarahm 2020). Changes in vegetation productivity and composition may, in turn, affect the distribution of forage, shelter, and denning habitat used by black bears. Likewise, temperature helps determine growing conditions, vegetation zones, and seasonal habitat characteristics, all of which can influence the spatial distribution of suitable habitat. The importance of precipitation and temperature in this study, therefore, likely reflects their role in shaping habitat quality through ecosystem productivity and forest composition. This interpretation is consistent with previous studies showing that climatic variables, particularly temperature and precipitation, are major determinants of species distributions and habitat suitability patterns (Bradie and Leung 2017; Hamid et al. 2019; Yuan et al. 2020). Similar patterns were identified in other regional studies, where mean temperature, precipitation, and elevation emerged as key bioclimatic determinants of black bear distribution (Farashi and Erfani 2018; Deb et al. 2019; Rehan et al. 2024).

Forest cover played a critical role in determining black bear habitat. The species showed higher suitability in mixed and heterogeneous forests, consistent with findings from across the Himalayan and East Asian ranges (Bista and Aryal 2013; Takahata et al. 2013). Black bears are opportunistic omnivores, using multiple land‐cover types where food is seasonally available (Kichloo and Sharma 2021). Habitat suitability showed a non‐linear relationship with human presence. Suitability increased with human population up to relatively high values, but declined at the highest population levels, suggesting that black bears may use moderately populated forest‐settlement interface areas while avoiding more intensively disturbed landscapes. A similar pattern was observed for settlement proximity: suitability increased near settlements, which likely reflects the availability of forest patches, croplands, livestock areas, orchards, and other human‐derived food resources around settlement edges. Comparable patterns have been reported from Bhutan and India, where black bears have been observed near villages and settlements, particularly in landscapes containing croplands and other anthropogenic food sources (Sathyakumar 2001; Charoo et al. 2011; Letro et al. 2020). In Bhutan, bear sightings also increased during crop‐production periods, indicating seasonal attraction to cultivated areas (Jamtsho and Wangchuk 2016). Previous studies further show that fragmented habitats adjoining settlements, croplands, and orchards can increase the likelihood of close human–bear interactions (Bargali et al. 2005). The pattern observed in the present study, therefore, likely reflects black bear use of mixed‐use edge landscapes in the Gaurishankar Conservation Area, while the decline at the highest human population values suggests avoidance of areas with intense human disturbance. However, habitat suitability declined with increasing proximity to roads, highlighting the species' avoidance of heavily fragmented or disturbed areas. Similar ecological responses have been observed in studies from Makalu Barun National Park, where black bears favoured temperatures of 10°C–20°C, moderate precipitation, and proximity to forest edges (Su et al. 2021). Across the Himalayas, seasonal food availability is known to drive black bear movement along altitudinal gradients, with individuals migrating between elevations to exploit changing fruit and nut availability (Izumiyama and Shiraishi 2004; Sathyakumar and Choudhury 2007; Wang et al. 2008; Hwang et al. 2010).

Understanding residents' perspectives on wildlife is essential for successful management and conflict reduction (Best and Pei 2020), as it helps gauge community acceptance of protected species, understanding of conservation measures, and overall support for conservation initiatives (Kideghesho et al. 2007). The majority of respondents reported that human‐black bear interactions and conflicts are increasing, which aligns with earlier findings in the same region (Pathak et al. 2024). Black bears are known to depredate livestock and raid crops (Chauhan 2003; Charoo et al. 2011), both in sheds and during grazing. Similar trends of increasing conflicts have been documented in the Annapurna and Manaslu Conservation Area (Bista and Aryal 2013; Chetri 2013). Our results indicate that negative attitudes toward black bears persist, largely shaped by past experiences of livestock attacks, crop raids, and occasional human casualties, despite general support for black bear conservation (Ali et al. 2018). Crop damage and livestock losses caused by wildlife impose high economic costs on local farmers, often leading to unfavourable perceptions of wildlife and conservation efforts (Priston and Underdown 2009; Ango et al. 2017). Key factors influencing conflict perception in the GCA include gender, elevation, ethnicity, recognition of ecological value, and perceived threats to crops and livestock. A high Pseudo *R* ^2^ value suggests the model explained a substantial proportion of variability in perceptions (Issa and Kogan 2014). The highly significant likelihood ratio *χ* ^2^ test (*p* = 0.0000064) confirms that the explanatory variables collectively had a significant effect on perceived conflict.

The negative coefficient for male respondents indicates that men were less likely than women to perceive an increase in human–black bear conflict. Conversely, female respondents were more likely to report increasing conflict, which may reflect their greater involvement in forest‐edge activities such as fuelwood collection, fodder gathering, agriculture and livestock care. This pattern is consistent with findings from a human‐wildlife conflict study in Namibia, which showed that women were more vulnerable to wildlife‐related conflict than men and faced greater difficulties obtaining compensation and recovering from associated losses (Khumalo and Yung 2015). Earlier research in Nepal found that men were more likely to participate in wildlife tourism, potentially leading to more positive perceptions (Bista and Aryal 2013). This also aligns with earlier findings that gender can influence attitudes toward conservation (Büscher and Ramutsindela 2016). Since women often engage in fuelwood collection and agriculture near forest edges, they may experience more direct encounters with black bears, explaining their higher perception of conflict. Nepal's socio‐economic structure places women at the forefront of natural resource management; they play a central role in using and managing forests, water, agriculture, livestock, and fisheries (Upadhyay 2005). Women account for more than 60% of the agricultural workforce (Pradhan 1985; Food and Agriculture Organization of the United Nations 2011) and are chiefly responsible for gathering fuelwood and fodder for household needs (Acharya and Lynn 1982). This heavy dependence on natural resources increases women's exposure to wildlife and their vulnerability to conflict incidents. Similar gender‐based differences have been observed in other Himalayan regions (Khadka and Nepal 2010; Bhattarai and Fischer 2014). Similarly, Khumalo and Yung (2015) documented comparable gendered patterns, noting that women bore a heavier burden from human‐wildlife conflict in Namibia, a pattern that may also be relevant in Nepal. Similarly, in many male‐dominated societies, women often have limited opportunities to engage meaningfully in conservation initiatives due to structural and cultural constraints (Lendelvo et al. 2012). Gender and social inequalities are evident in both how impacts are distributed and the ability to cope with them. Women often face greater exposure to risks due to their involvement in forest product collection, yet they typically have more limited access to resources needed to adopt protective measures (Silwal et al. 2013).

The ethnic composition of respondents was dominated by Indigenous groups, who tend to depend heavily on forest resources for their livelihoods (Awasthi and Singh 2015). This greater dependence may increase the frequency of human‐black bear encounters, thereby explaining the higher perceived conflict between these groups. Previous studies also demonstrate this finding (Allendorf et al. 2007; Spiteri and Nepal 2008; Thapa and Hubacek 2011; Karanth and Nepal 2012). However, this finding contrasts with studies from other protected areas in Nepal, where Indigenous communities generally exhibit more positive attitudes toward conservation. For instance, in Langtang (LNP) and Bardiya National Parks (BNP), groups such as the Tamang, Hyolmo, and Tharu benefit from park‐related tourism and resources, which likely promotes more favourable views toward wildlife (Thapa et al. 2025).

Finally, the positive relationship between elevation and perceived conflict suggests that households at higher elevations, located closer to dense forests and suitable black bear habitats, experience more frequent interactions and thus perceive higher levels of conflict. Communities located farther from protected areas or forest corridors tend to hold more favourable views toward conservation (Shrestha and Alavalapati 2006; Badola et al. 2021), whereas those residing close to protected area boundaries often express negative perceptions of wildlife and conservation initiatives (Ochieng et al. 2021). This pattern likely arises because people living at greater distances experience fewer direct conflicts or negative impacts from protected areas, while those nearby face more frequent challenges (Mackenzie and Ahabyona 2012; Prins et al. 2022). Other variables such as education, age, human injury/death, distance, livestock unit, landholdings, number of bears seen, and time did not show any significant correlation.

This study has several limitations. For instance, it was not possible to collect occurrence data for the black bear across its entire GCA range, largely due to the region's rugged mountainous terrain and severe climatic conditions. Limited availability of high‐resolution environmental data across Nepal constrains both the model's accuracy and its applicability to other regions. Also, limitations should be considered when interpreting the habitat suitability map. The model‐based representation of habitat suitability should not be interpreted as direct evidence of occupancy across the entire landscape (Yackulic et al. 2013), since only occurrence locations recorded during field surveys were empirically confirmed. As with any predictive model, uncertainty remains, particularly for a flexible and wide‐ranging species such as the black bear (Jiménez‐Valverde et al. 2008). In addition, the vegetation predictor was represented by a categorical raster that may not fully capture finer ecological differences among forest types across complex mountainous terrain. Although these limitations may affect fine‐scale interpretation, the model remains useful for identifying broad patterns of suitability and potential zones of human‐bear interaction. On the other hand, the social survey findings should also be interpreted with caution because perception‐based responses are shaped not only by direct experience of conflict, but also by respondents' emotions, fear, prior experiences, and wider social context (Johansson et al. 2016; Castillo‐Huitrón et al. 2020). We also acknowledge that the approach we followed may introduce recall bias and overstatement of conflict severity (Naughton‐Treves et al. 2003). In addition, self‐reported attitudes and perceptions do not necessarily provide a purely objective measure of conflict severity, as such responses may vary across individuals depending on psychological disposition, question interpretation, and local circumstances (Basak et al. 2023). The questionnaire results should therefore be understood as socially situated perceptions of human‐black bear interactions rather than as exact measures of ecological risk alone.

## 5 Conclusion

This study provides the first comprehensive assessment of habitat suitability and socioeconomic drivers of human‐black bear conflicts in the GCA. Results indicate that the black bear distribution is shaped by both environmental and anthropogenic factors. Elevation, precipitation, and temperature were the strongest predictors, with bears favouring low‐ to mid‐elevation temperate forests (1000–3000 m) characterised by structural diversity and year‐round moisture. Additionally, gender, ethnicity, elevation, ecological value of the bear, and perception of bears as threats significantly affected respondents' views toward wildlife conservation and their perception of increasing conflicts. Women and Indigenous communities perceived greater conflict risks due to more frequent encounters near forest edges. These insights highlight the importance of integrating community‐based awareness, education, and livelihood interventions into conservation strategies to foster coexistence. The habitat suitability map generated through MaxEnt provides a valuable spatial framework for identifying priority conservation zones, potential habitat corridors, and areas of high human‐black bear interaction risk. Such information can guide land‐use planning, targeted monitoring, and the design of conflict mitigation strategies, particularly in zones with high habitat suitability near human settlements.

## Author Contributions

**Shreyashi Bista:** conceptualization (lead), data curation (lead), formal analysis (lead), funding acquisition (lead), investigation (lead), methodology (lead), writing – original draft (lead), writing – review and editing (lead). **Nani Raut:** supervision (equal), writing – review and editing (supporting). **Suman Shree Neupane:** data curation (equal), formal analysis (supporting), writing – review and editing (equal). **Morten Odden:** supervision (lead), writing – review and editing (lead). **Madhu Chetri:** conceptualization (equal), funding acquisition (equal), methodology (equal), supervision (lead), writing – review and editing (lead).

## Funding

This work was supported by Idea Wild (BISTNEPA1022‐00) and Rufford Foundation (38121‐1, 32168‐1).

## Conflicts of Interest

The authors declare no conflicts of interest.

## Supporting information


**Data S1:** ece373776‐sup‐0001‐DataS1.docx.

## Data Availability

All data supporting the findings of this study, including species presence points, environmental variables used for modelling, perception survey data, R scripts for ecological and statistical analyses, and the Wallace RDS file, are provided as Supporting Information with this submission. These files have been made available to editors and reviewers for the peer‐review process and will be deposited in the Zenodo public repository upon acceptance of the manuscript. DOI: https://doi.org/10.5281/zenodo.20365116.
