# Correction to “Ecological and Social Dimensions of Human–Bear Coexistence in Nepal's Gaurishankar Conservation Area”

**DOI:** 10.1002/ece3.73985

**Published:** 2026-07-03

Bista, S. , N. Raut , S. S. Neupane , M. Odden , and M. Chetri . 2026. “Ecological and Social Dimensions of Human–Bear Coexistence in Nepal's Gaurishankar Conservation Area.” Ecology and Evolution 16, no. 6: e73776. 10.1002/ece3.73776.42256129 PMC13239942

In Table 4, the values associated with the terms “Decreased” and “Increased” are swapped. The value 85.88% should be associated with “Increased,” while the value 5.88% should be associated with “Decreased.” The corrected Table 4 is provided below.

**TABLE 4:** Socio‐economic characteristics of surveyed respondents (*n* = 188) in Bigu Rural Municipality, Gaurishankar Conservation Area, Nepal.

Socio‐economic indicators	Values
Conflict level	
Decreased	5.88%
Same	8.24%
Increased	85.88%
Gender	
Male	47%
Female	53%
Ethnicity	
Indigenous	43%
Bramhin/Chhetri/Thakuri	32%
Dalit	25%
Education	
Uneducated	49%
Primary education	26 %
Secondary	20%
Higher secondary	4%
University	1%
Age	38.78 ± 10.58 (18–68)
Landholdings (in hectares)	0.41 ± 0.36 (0.05–3.1)
Livestock unit (LSU)	8.82 ± 7.65 (0–47)
Bear seen	
Yes	60%
No	40%
Number of bears seen	1.37 ± 0.69 (1–5)
Time	5.6 ± 4.69 (0–16)
Distance	200.1913

In the subsequent paragraph of Table 4, the corresponding value has been rounded off. The fourth sentence should read as follows for consistency with the value in Table 4:

Regarding conflict, **85.88%** reported an increase, **8.24%** a decrease, and **5.88%** had no opinion, indicating that local people may be experiencing growing conflict in the study area (Table 4).

We apologize for this error.

