# Supplementary material for: Ecological and Social Dimensions of Human–Bear Coexistence in Nepal's Gaurishankar Conservation Area
Source: Ecol Evol. 2026 May 31;16(6):e73776. doi: 10.1002/ece3.73776 (PMC13239942; doi:10.1002/ece3.73776)
Supplement: Supplementary file 1 — Data S1: ece373776‐sup‐0001‐DataS1.docx. [file ECE3-16-e73776-s001.docx]

**Supplementary**

Questionnaires for conflict data collection and perception study

1. DEMOGRAPHIC INFORMATION
2. Rural Municipality:
3. Ward number:
4. Village name:
5. Education:
6. Ethnicity:
7. Age:
8. Major Occupation:
9. Sex: M/F

AGRICULTURE CROP DATA AND LIVESTOCK INFORMATION

10 Total Agricultural farmland- (in Ropani/hectares) ……………………….

| S.N. | Name of Crop | Production-2023 | Loss-2023 | Month (germination to  harvesting) |
| --- | --- | --- | --- | --- |
| 1. | Maize |  |  |  |
| 2. | Millet |  |  |  |
| 3. | Potatoes |  |  |  |
| 4. | Wheat |  |  |  |
| 5. | Rice |  |  |  |
|  | Others……. |  |  |  |
|  |  |  |  |  |
|  |  |  |  |  |
|  |  |  |  |  |

**LIVESTOCK AND DEPREDATION INFORMATION**

1. What types of livestock are primarily targeted by wild carnivores (e.g., goats, oxen, sheep, yak)?
2. Which species of wild carnivores are responsible for these above losses?
3. Which livestock are most targeted by ABB?

14 Have your livestock been depredated by ABB?

i)Yes ii) No

15. Method of verification………….

INFORMATION ON ABB AND ITS LOCATION

16. Have you seen ABB?

Yes b. No

17. Year/Month………………………

18. Number…………………….

19. If yes, where did the encounter take place?

Forest area b. Farmland/Agricultural land c. Others/……… (specify)

20. If, forest area, (specify name):

21. Forest species: ………………………………………………………

22. If village, distance to nearest forest…………….

23. At what time of the day did the encounter take place?

Morning (3 AM-6PM) b. Day (6 AM-6 PM) c. Evening (6 PM-9PM) d. Night (9 PM- 3AM)

24. Feeding species (wild) of ABB e.g.: (tusa, baanko, oak-khasru)

25. What was the most preferred wild fruit by ABB?........................

26. Name of the crops that ABB feeds on……………………………………………

27. What was the crop most raided by black bear?................................

28. What were the mitigation measures applied by local people to prevent black bear conflict?

29. What kind of techniques do you apply on encountering a black bear?

a. Shouting b. Chasing with fire

c. Drumming utensils d. No action e. Using guard dogs

30. Has there been any settlement shifting due to the bear attack?

a. Yes b. No c. Don’t know

31. Have you left some land area fallow in the last 2 years due to the problem caused by the Black Bear?

a. Yes b. No c. Don’t know

HUMAN INJURIES:

32. Have there been any injuries/deaths from animal attacks in your locality?

a. Yes b. No c. Don’t know

COMPENSATION:

33.Have you been compensated for human attack or casualty?

a. Yes b. No c. Don’t know

If yes, how much……………………….

34. Have you been compensated for livestock depredation?

a. Yes b. No c. Don’t know

If yes, how much……………………….

35. Are farmers compensated for the damage done by crop-raiding bears?

a. Yes b. No c. Don’t know

If yes? How much……………………………………….

36. Are you satisfied with the compensation given to you?

a. Yes b. No c. Don’t know

LOCAL PERCEPTION OF ABB:

37. Why do you think the bear comes out from the forest?

a. Lack of food in the forest b. Habitat Degradation

c. Seasonal Migration d. Others……... (Increment in bear population)

38. Does black-bear have any ecological value?

Yes b. No c. Don’t know

39. Do you think black-bear should be conserved?

Yes b. No c. Don’t know

40. Do you think black bear is threat to your crop and livestock?

Yes b. No c. Don’t know

41. What do you think about the status of Human-Bear conflict?

a. Increasing b. Decreasing c. Don’t know

42. If yes, what should be done to mitigate the human-bear conflict?

1. Removing bears c. Avoid risk areas
2. Guarding for bears (Fencing) d. Resettle e. Deforestation f. Don’t know
